# The Histamine 3 Receptor Is Expressed in the Heart and Its Activation Opposes Adverse Cardiac Remodeling in the Angiotensin II Mouse Model

**DOI:** 10.3390/ijms21249757

**Published:** 2020-12-21

**Authors:** Samuel L. McCaffrey, Grace Lim, Martyn Bullock, Ainsley O. Kasparian, Roderick Clifton-Bligh, William B. Campbell, Alexander Widiapradja, Scott P. Levick

**Affiliations:** 1 Kolling Institute for Medical Research, Royal North Shore Hospital, St Leonards, NSW 2064, Australia; sam.mccaffrey15@gmail.com (S.L.M.); e.lim@sydney.edu.au (G.L.); martyn.bullock@sydney.edu.au (M.B.); ainsley.kasparian@sydney.edu.au (A.O.K.); roderick.cliftonbligh@sydney.edu.au (R.C.-B.); Alexander.widiapradja@sydney.edu.au (A.W.); 2 Faculty of Medicine and Health, The University of Sydney, Camperdown, NSW 2006, Australia; 3 School of Life Sciences, University of Technology Sydney, Ultimo, NSW 2007, Australia; 4 Department of Pharmacology and Toxicology, Medical College of Wisconsin, Milwaukee, WI 53226, USA; wcamp@mcw.edu

**Keywords:** mast cell, macrophage, fibroblast, imetit, A331440, histamine receptor agonist, histamine receptor antagonist, collagen, fibrosis, extracellular matrix

## Abstract

Histamine is a basic amine stored in mast cells, with its release capable of activating one of four histamine receptors. The histamine 3 receptor (H_3_R) is known to be cardioprotective during acute ischemia by acting to limit norepinephrine release. However, a recent study reported that myofibroblasts isolated from the infarct zone of rat hearts responded to H_3_R activation by up-regulating collagen production. Thus, it is necessary to clarify the potential role of the H_3_R in relation to fibrosis in the heart. We identified that the mouse left ventricle (LV) expresses the H_3_R. Isolation of mouse cardiac fibroblasts determined that while angiotensin II (Ang II) increased levels of the H_3_R, these cells did not produce excess collagen in response to H_3_R activation. Using the Ang II mouse model of adverse cardiac remodeling, we found that while H_3_R blockade had little effect on cardiac fibrosis, activation of the H_3_R reduced cardiac fibrosis and macrophage infiltration. These findings suggest that when activated, the H_3_R is anti-inflammatory and anti-fibrotic in the mouse heart and may be a promising target for protecting against cardiac fibrosis.

## 1. Introduction

Adverse myocardial remodeling, including cardiac fibrosis and cardiomyocyte hypertrophy, can lead to LV diastolic and systolic dysfunction and eventual heart failure. There is substantial evidence to support a contribution of mast cells to cardiac fibrosis in particular, through the release of pre-stored mediators such as the proteases tryptase and chymase [1,2,3,4,5,6,7,8,9]. Histamine, which is derived from the amino acid histidine via decarboxylation by the enzyme histidine decarboxylase, is another pre-stored mast cell product. Upon its release, histamine can bind to any of four G-protein coupled histamine receptors. These are the histamine 1 receptor (H_1_R), histamine 2 receptor (H_2_R), H_3_R, and histamine 4 receptor (H_4_R). Of these four histamine receptors, there is already strong evidence supporting a role for the H_2_R in the development of cardiac fibrosis. The H_2_R is expressed by cardiac fibroblasts, and its activation can stimulate a pro-fibrotic phenotype in isolated mouse cardiac fibroblasts [10]. H_2_R deletion in mice led to reduced cardiac fibrosis and improved cardiac function following aortic banding. Clinically, the Multi-Ethnic Study of Atherosclerosis (MESA) trial reported that patients receiving H_2_R antagonist treatment for gastric acid production had a 62% lower risk of developing heart failure [11].

Conversely, the H_3_R has traditionally been associated with cardioprotection. Seminal work from Roberto Levi’s group demonstrated that the H_3_R is localized to presynaptic cardiac sympathetic nerve fibers and, when activated during acute ischemia, acts to limit norepinephrine release [12,13,14,15]. The functional outcome of H_3_R activation with imetit during acute ischemia-reperfusion was a reduction in the incidence of ventricular fibrillation and arrhythmias [16]. However, a recent article by Piera et al. [17] reported that myofibroblasts isolated from the infarct zone of rat hearts following four weeks of ischemia responded to the H_3_R agonist imetit by up-regulating collagen production. This indicated a possible long-term pro-fibrotic role for the H_3_R. Importantly though, this study only investigated isolated fibroblasts and did not establish an in vivo role for the H_3_R in cardiac fibrosis. Thus, there is a need to clarify the contribution, if any, of the H_3_R to cardiac fibrosis in vivo. Accordingly, we used the Ang II-infused mouse model to investigate the role of the H_3_R in cardiac fibrosis. We found that (1) the H_3_R is expressed in the mouse LV; (2) isolated cardiac fibroblasts possess the H_3_R, which was up-regulated in response to Ang II; (3) blockade of the H_3_R with A331440 did not alter cardiac fibrosis or inflammation; and (4) activation of the H_3_R with imetit significantly reduced cardiac fibrosis and macrophage infiltration.

## 2. Results

### 2.1. The H_3_R Is Expressed in the Mouse Heart

**Figure 1:**
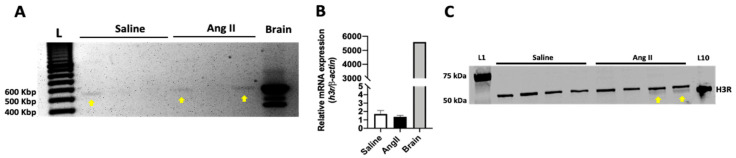
The mouse heart expresses the H_3_R. (**A**) PCR identified a ~530 bp product for the saline and Ang II mouse LV (yellow arrows). (**B**) real-time PCR quantification of H_3_R mRNA from hearts of saline and Ang-II-infused mice (*n* = 5/group). The brain is the positive control (*n* = 1). Data are mean ± SEM. (**C**) Western blotting identifying the presence of H_3_R protein (yellow arrows) only in hearts from mice infused with Ang II and not from saline-infused mice. L1 = ladder, L10 = brain as positive control.

We first determined whether the H_3_R was expressed in the mouse heart, as well as the number of isoforms expressed. For the positive control brain, PCR revealed the expected three products corresponding to the ~530, 430, and 400 bp isoforms of the H_3_R as described previously [18] (Figure 1A and Figure S1). However, the mouse LV expressed only the 530 bp isoform. Furthermore, a PCR product was not detected for every LV. Sanger sequencing of the 530 bp product revealed a 99% match for the mouse H_3_R in both the LV and brain (Figures S2 and S3A,B). A 200 bp product observed in the LV samples was also sequenced and identified as endoplasmic reticulum chaperone SIL1 homolog (Figures S2 and S3C,D). Using real-time PCR, we quantified H_3_R LV expression levels for saline and Ang II groups. Due to the more sensitive nature of real-time PCR, we were able to detect product for the H_3_R in all hearts and found no difference in H_3_R expression levels between saline and Ang II groups (Figure 1B). We then investigated H_3_R protein in saline and Ang II hearts. Western blotting revealed two bands at, and slightly above, the predicted 50 kDa molecular weight for the H_3_R protein in the Ang II heart but only the larger product for the saline heart (Figure 1C and Figure S4). Using the brain as a positive control and indicator of the correct molecular weight, we concluded that the lower band of the doublet represented the actual H_3_R, and the upper band was non-specific binding. Therefore, while mRNA levels were equal between the two groups, it appeared that the H_3_R is translated to protein only in Ang II hearts.

### 2.2. Ang II Increases H_3_R Levels in Isolated Cardiac Fibroblasts

**Figure 2:**
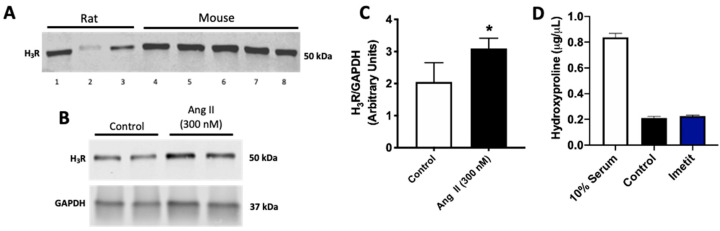
Isolated cardiac (myo)fibroblasts express the H_3_R. (**A**) Western blot for the H_3_R in isolated rat and mouse cardiac (myo)fibroblast lysate. Cardiac fibroblasts were exposed to varying culture conditions designed to maintain a more quiescent fibroblast phenotype or to induce myofibroblast conversion to determine under which conditions the H_3_R was present: Lane 1 = rat fibroblasts grown on plastic, Lane 2 = rat fibroblasts grown on gelatin, Lane 3 = rat fibroblast in high serum (10% serum), Lane 4 = mouse fibroblasts grown on gelatin, Lane 5 = mouse fibroblasts in 10% serum, Lane 6 = mouse fibroblasts treated with Ang II (300 nM), Lane 7 = mouse fibroblasts treated with TGF-β1 (10 ng/mL), and Lane 8 = mouse fibroblasts in high glucose (25 mM). (**B**) Western blot for the H_3_R in isolated mouse cardiac fibroblasts treated with Ang II (300 nM). GAPDH served as the loading control. (**C**) quantification of the H_3_R in isolated mouse cardiac fibroblasts treated with Ang II (*n* = 4/group). (**D**) Hydroxyproline as a marker of collagen production in the media from isolated mouse cardiac fibroblasts treated with the H_3_R agonist, imetit (100 nM, *n* = 6/group). 10% serum served as a positive control. Data are mean ± SD (**C**) or SEM (**D**). * = *p* < 0.05 vs. Control.

Recent literature suggests that the H_3_R is expressed on isolated rat cardiac myofibroblasts and that its activation may be involved in the positive regulation of collagen production by these cells [17]. We sought to determine whether the presence of the H_3_R was restricted to the myofibroblast phenotype. Accordingly, we sought to determine whether culture conditions influencing fibroblast phenotype could alter H_3_R expression. We cultured isolated rat and mouse cardiac fibroblasts under various conditions designed to maintain the quiescent fibroblast phenotype or to promote a myofibroblast phenotype. Western blotting identified the H_3_R on isolated cardiac fibroblasts and myofibroblasts under all conditions tested (Figure 2A, see Figure Legend for culture condition details, Figure S5). Subsequently, we treated isolated mouse cardiac fibroblasts with Ang II, which significantly increased the amount of H_3_R on those cells (*p* < 0.05, Figure 2B,C and Figure S6). However, while the 10% serum positive control increased hydroxyproline as a surrogate for collagen production, treatment of isolated mouse cardiac fibroblasts with the H_3_R agonist imetit did not increase the production of hydroxyproline by these cells (*p* = 0.32 vs. Control, Figure 2D).

### 2.3. Effect of H_3_R Blockade on LV Structure and Function in Ang-II-Infused Mice

**Figure 3:**
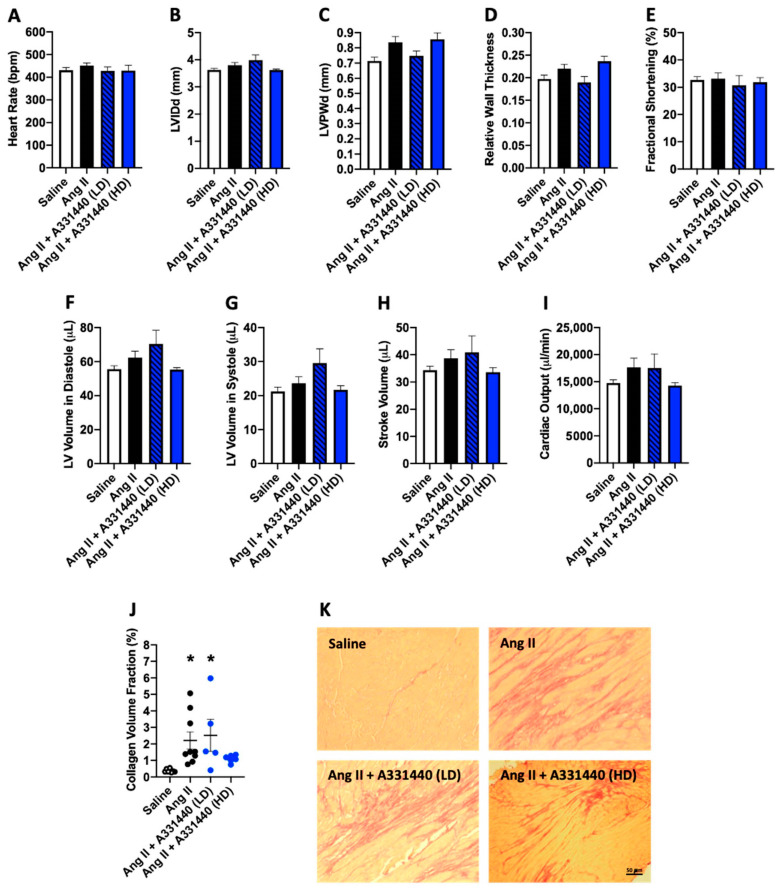
Blockade of the H_3_R does not prevent adverse cardiac remodeling. Echocardiographic assessment of heart rate (**A**); left ventricular internal diameter in diastole (LVIDd, (**B**)); left ventricular posterior wall thickness in diastole (LVPWd, (**C**)); relative wall thickness (**D**); fractional shortening (**E**); left ventricular volume in diastole (**F**); left ventricular volume in systole (**G**); stroke volume (**H**); cardiac output (**I**); left ventricular collagen volume fraction as a marker of fibrosis in saline, Ang II, Ang II + low-dose (LD) A331440, and Ang II + high-dose (HD) A331440 mice (**J**); representative picrosirious red stained images used to determine collagen volume fraction (**K**). All data are mean ± SEM. Saline, *n* = 8; Ang II, *n* = 9; Ang II + A331440 (LD), *n* = 5; Ang II + A331440 (HD), *n* = 6; * = *p* < 0.05 vs. saline.

Although H_3_R activation did not induce a pro-fibrotic phenotype for isolated cardiac fibroblasts, for completeness we tested the effect of H_3_R blockade in vivo. Table 1 displays the biometric data for mice treated with the H_3_R antagonist A331440. Body weight was not different between untreated saline and Ang II mice. Low-dose A331440 (10 mg/kg/day) did not alter body weight, however, body weight was significantly lower in the high-dose A331440 group (20 mg/kg/day, *p* < 0.01 vs. saline, *p* < 0.0001 vs. Ang II, *p* < 0.001 vs. Ang II + A331440 LD). Ang II caused a significant increase in LV mass (*p* < 0.001) as well as the LV/tibia ratio (*p* < 0.01) compared to the saline group. There was a non-significant trend for LV mass to also be increased in Ang II mice receiving low-dose A331440 (*p* = 0.15 vs. saline, *p* = 0.99 vs. Ang II); the same trend existed for the LV/tibia ratio (*p* = 0.15 vs. saline, *p* = 0.97 vs. Ang II). LV mass was normalized by treatment with high-dose A331440 (*p* < 0.01 vs. Ang II, *p* < 0.001 vs. Ang II + A331440 LD, *p* = 0.99 vs. saline), as was the LV/tibia ratio (*p* < 0.01 vs. Ang II, *p* < 0.001 vs. Ang II + A331440 LD, *p* = 0.99 vs. saline). There were no significant differences between any of the groups for right ventricular (RV) mass, lung mass, RV/tibia, or lung/tibia ratios. While, generally speaking, there were no significant differences in the echocardiography parameters assessed (Figure 3A–I and Figure S7), there was a strong trend for LVPWd to be increased in Ang II (*p* = 0.07 vs. saline) and high-dose Ang II + A331440 groups (*p* = 0.06 vs. saline, Figure 3C). There were no differences in functional parameters between groups (Figure 3E,H,I).

### 2.4. H_3_R Blockade Did Not Alter Cardiac Fibrosis

Next, we examined whether H_3_R blockade altered cardiac fibrosis. Ang II increased collagen volume fraction in the LV compared to saline controls, indicative of fibrosis (Figure 3J,K, *p* < 0.05). Neither low-dose nor high-dose H_3_R antagonist A331440 significantly reduced cardiac fibrosis compared to Ang-II-infused mice (*p* = 0.91 low-dose A331440 vs. Ang II; *p* = 0.11 high-dose A331440 vs. Ang II).

### 2.5. H_3_R Blockade Did Not Alter Inflammation

**Figure 4:**
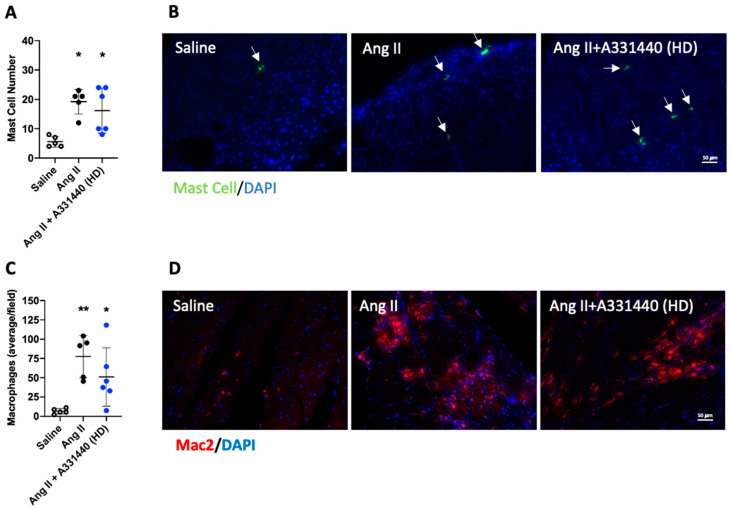
Cardiac mast cell and macrophage numbers in response to H_3_R blockade with A331440. (**A**) quantification of mast cells/LV section; (**B**) representative images for cardiac mast cells as indicated by white arrows (green = mast cell, blue = DAPI); (**C**) quantification of macrophages/LV field; (**D**) representative images for cardiac macrophages (red = macrophages, blue = DAPI). All data are mean ± SD. Saline, *n* = 5; Ang II, *n* = 5; Ang II + A331440, *n* = 6; * = *p* < 0.05 vs. saline, ** = *p* < 0.01 vs. saline.

We assessed the number of mast cells and macrophages in the heart to determine whether H_3_R blockade altered inflammation in the heart. The number of mast cells was significantly increased in Ang-II-infused hearts (*p* < 0.05 vs. saline, Figure 4A,B). High-dose H_3_R blockade with A331440 did not alter the increase in mast cells induced by Ang II (*p* < 0.05 vs. saline; *p* = 0.63 vs. Ang II). The number of cardiac macrophages was also significantly increased in Ang-II-infused mice (*p* < 0.01 vs. saline, Figure 4C,D). Macrophage numbers were also significantly increased in mice treated with high-dose A331440 (*p* < 0.05 vs. saline; *p* = 0.29 vs. Ang II).

### 2.6. Effect of H_3_R Activation on LV Structure and Function in Ang-II-Infused Mice

**Figure 5:**
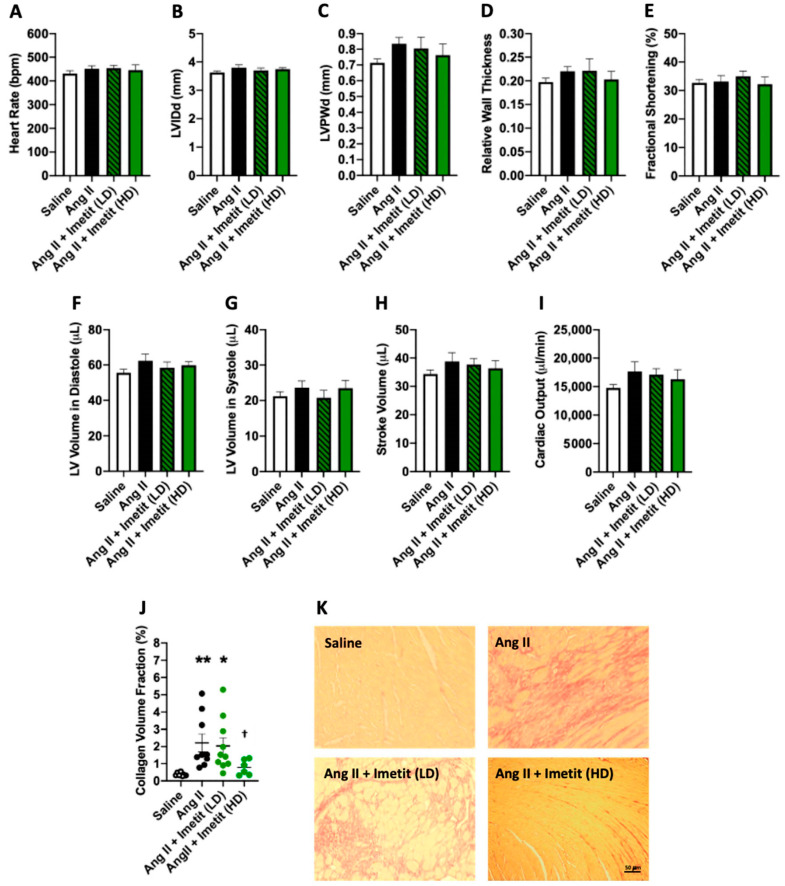
Activation of the H_3_R with imetit improves cardiac remodeling. (**A**) heart rate; (**B**) left ventricular internal diameter in diastole (LVIDd); (**C**) left ventricular posterior wall thickness in diastole (LVPWd); (**D**) relative wall thickness; (**E**) fractional shortening; (**F**) left ventricular volume in diastole; (**G**) left ventricular volume in systole; (**H**) stoke volume; (**I**) cardiac output; (**J**) left ventricular collagen volume fraction as a marker of fibrosis in saline, Ang II, Ang II + low-dose (LD) imetit, and Ang II + high-dose (HD) imetit mice; (**K**) representative picrosirious red stained images used to determine collagen volume fraction. All data are mean ± SEM. Saline, *n* = 8; Ang II, *n* = 9; Ang II + imetit (LD), *n* = 8; Ang II + imetit (HD), *n* = 6; * = *p* < 0.05 vs. saline, ** = *p* < 0.01 vs. saline, † = *p* < 0.05 vs. Ang II.

Having determined that H_3_R blockade did not alter adverse cardiac remodeling, we then investigated whether H_3_R activation could improve remodeling. Table 1 displays the biometric data for mice treated with the H_3_R agonist imetit. Body weight was not different between untreated saline and Ang II mice. Low-dose imetit (20 mg/kg/day) did not alter body weight, however, body weight was significantly lower in the high-dose imetit group (40 mg/kg/day, *p* < 0.05 vs. saline, *p* < 0.0001 vs. Ang II, *p* < 0.01 vs. Ang II + imetit LD). Ang II caused a significant increase in LV mass (*p* < 0.001) as well as the LV/tibia ratio (*p* < 0.01) compared to the saline group. LV mass was also significantly increased in Ang II mice receiving low-dose imetit (*p* < 0.001 vs. saline, *p* = 0.99 vs. Ang II), as was the LV/tibia ratio (*p* < 0.001 vs. saline, *p* = 0.97 vs. Ang II). LV mass was normalized by treatment with high-dose imetit (*p* < 0.01 vs. Ang II, *p* < 0.001 vs. Ang II + imetit LD, *p* = 0.99 vs. saline), as was the LV/tibia ratio (*p* < 0.01 vs. Ang II, *p* < 0.001 vs. Ang II + imetit LD, *p* = 0.99 vs. saline). There were no significant differences between any of the groups for RV mass, lung mass, RV/tibia, or lung/tibia ratios. While, generally speaking, there were no significant differences in the echocardiography parameters assessed (Figure 5A–I and Figure S7), high-dose imetit tended to reduce LVPWd (Figure 5C) and hence relative wall thickness (Figure 5D). There were no differences in functional parameters between groups (Figure 5E,H,I).

### 2.7. H_3_R Activation Does Alter Cardiac Fibrosis

Ang II increased the collagen volume fraction in the LV compared to saline controls, indicative of fibrosis (Figure 5J,K and *p* < 0.01). Ang-II-infused mice treated with low-dose H_3_R agonist imetit also had increased collagen volume fraction compared to saline mice (*p* < 0.05 vs. saline; *p* = 0.74 vs. Ang II). Conversely, high-dose imetit significantly reduced collagen volume fraction (*p* < 0.05 vs. Ang II).

### 2.8. Norepinephrine Levels in the Heart

Ang II infusion did not alter cardiac norepinephrine levels at the time point examined in this study (Figure S8, *p* = 0.69 vs. saline). Due to the lack of effect of Ang II on norepinephrine at this time point, we did not assess the effects of high-dose imetit.

### 2.9. H_3_R Activation Reduced Inflammation

**Figure 6:**
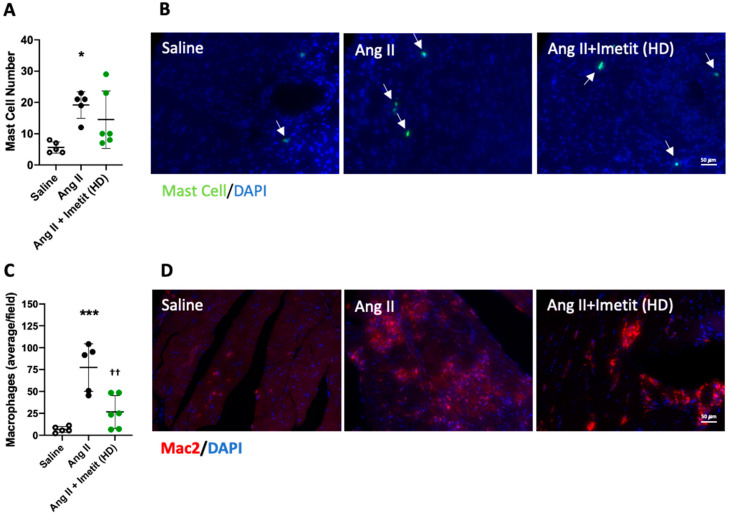
Cardiac mast cell and macrophage numbers in response to H_3_R activation with imetit. (**A**) quantification of mast cells/LV section; (**B**) representative images for cardiac mast cells as indicated by white arrows (green = mast cell, blue = DAPI); (**C**) quantification of macrophages/LV field; (**D**) representative images for cardiac macrophages (red = macrophages, blue = DAPI). All data are mean ± SD. Saline, *n* = 5; Ang II, *n* = 5; Ang II + imetit, *n* = 6; * = *p* < 0.05 vs. saline, *** = *p* < 0.001 vs. saline, †† = *p* < 0.01 vs. Ang II.

The number of mast cells in the LV was significantly increased in Ang-II-infused mice (*p* < 0.05 vs. saline, Figure 6A,B). There was a non-significant trend for mast cell numbers to be decreased in Ang-II-infused mice treated with high-dose imetit (*p* = 0.08 vs. saline, *p* = 0.45 vs. Ang II). The number of cardiac macrophages was also significantly increased in Ang-II-infused mice (*p* < 0.001 vs. saline, Figure 6C,D). Macrophage numbers were lower in mice treated with high-dose imetit compared to untreated Ang II mice (*p* < 0.01 vs. Ang II; *p* = 0.23 vs. saline).

## 3. Discussion

Our study identified the expression of the H_3_R in the mouse heart and established that activation of this receptor can counteract adverse cardiac remodeling induced by Ang II infusion. Specifically, we demonstrated that (1) the H_3_R was present in the mouse LV; (2) isolated cardiac fibroblasts possess the H_3_R, which was up-regulated in response to Ang II but did not induce a pro-fibrotic phenotype when activated; (3) blockade of the H_3_R with A331440 did not alter cardiac fibrosis or inflammation; and (4) activation of the H_3_R with imetit significantly reduced cardiac fibrosis, hypertrophy and macrophage infiltration.

We were able to identify mRNA expression of the H_3_R in the mouse LV. While PCR identified the three isoforms of the H_3_R known to be present in the rodent brain [18,19], we only detected one isoform in the mouse heart. This is in agreement with a previous report that the rat heart only expresses one H_3_R isoform [18]. Sanger sequencing of the 530 bp product confirmed it to be a 99% match for the H_3_R. The H_3_R had previously been identified on presynaptic cardiac sympathetic nerve fibers (cardiac synaptosomes) by pharmacological means [14]. The expression of mRNA isolated directly from the heart raises the question of which cells of the heart could express the H_3_R. We could not detect H_3_R protein in lysate from H9c2 cells (Figure S9), which are originally derived from rat atrial cardiomyocytes and now represent an immature cardiomyocyte phenotype. This, together with the low levels of the H_3_R, suggests that cardiomyocytes do not express the H_3_R. Recently, Piera et al. [17] reported that myofibroblasts isolated from the scar region of the rat heart four weeks post-ischemia possess the H_3_R and could increase collagen production in response to H_3_R activation with imetit. This was confirmed when the H_3_R antagonist ciproxifan reduced the increase in collagen production in response to histamine. Herein we confirmed that cardiac fibroblasts isolated from the normal mouse heart also possess the H_3_R, as well as when we cultured isolated rat and mouse cardiac fibroblasts under an array of conditions designed to allow comparison of fibroblast versus myofibroblast phenotype. Thus, cardiac (myo)fibroblasts, but not cardiomyocytes, are a likely source of the H_3_R in the heart. This may explain why H_3_R expression is low in the mouse heart since fibroblasts only account for roughly 15% of cells in the mouse heart [20]. Interestingly, we found that the H_3_R was increased on isolated cardiac fibroblasts when these cells were treated with Ang II, suggestive of a possible pro-fibrotic role. However, incubation of mouse cardiac fibroblasts with the H_3_R agonist imetit did not cause increased collagen production, which was in contrast to the findings of Piera et al. [17]. In the Piera et al. [17] study, what was termed “myofibroblasts” were fibroblasts isolated from the scar of the rat heart four weeks post-ischemia. It has recently been shown that fibroblasts residing in the infarct scar at chronic time points assume an intermediate phenotype coined the “matrifibrocyte” that is not a quiescent fibroblast but has lost the proliferative and α-smooth muscle actin characteristics typical of myofibroblasts [21]. Presumably, this was the phenotype of the fibroblasts isolated from the scar at four weeks post-infarct in the Piera et al. study. It may be possible that these matrifibrocytes respond differently to H_3_R activation. Furthermore, we also found that treatment of Ang-II-infused mice with the H_3_R antagonist, A331440, had no statistically significant effect on cardiac fibrosis in the Ang II model, indicating that the H_3_R is not pro-fibrotic in this model. In additional support of this finding, both mast cell and macrophage numbers remained elevated in the heart after A331440 treatment. Both mast cells and macrophages are important contributors to the cardiac fibrosis process [1,2,3,4,5,8,9,22,23,24].

In previous cardiac ischemia studies, activation of the H_3_R had been shown to be cardioprotective by down-regulating norepinephrine release. For example, activation of the H_3_R resulted in a reduction of norepinephrine release in an isolated guinea pig heart model of 20 min of ischemia followed by a 45-min reperfusion period [16]. Given the prominent role of norepinephrine in cardiac fibrosis [25], including Ang-II-induced cardiac fibrosis, we hypothesized that H_3_R activation could reduce fibrosis via down-regulation of norepinephrine. Herein we report that H_3_R activation with high-dose imetit significantly reduced cardiac fibrosis in the Ang II mouse model. Interestingly though, it is not clear whether this involved effects on norepinephrine as we did not observe up-regulation of norepinephrine by Ang II at the time point investigated in this study. However, we cannot rule out increases at earlier time points. There is some evidence that Ang II does not cause generalized norepinephrine release in the heart, at least in the pig [26]. That study used a micro-dialysis technique to measure norepinephrine in the cardiac interstitium, therefore it is possible that norepinephrine release is focal and was remote to the probe placement. Certainly, norepinephrine is important on some level in the Ang-II-infusion model since β-adrenergic receptor blockade prevents focal cardiomyocyte necrosis [27,28]. However, other mechanisms may be in play. Imetit was successful in reducing Ang-II-induced macrophage infiltration into the LV, which represents a mechanism by which H_3_R activation opposed fibrosis. Curiously, mast cell numbers were not significantly reduced by imetit, although, this was likely due to two of the six mouse hearts that remained considerably higher than the other four and led to a large standard deviation. Interestingly, we could not detect H_3_R protein on either bone-marrow-derived macrophages or mast cells (Figure S9), suggesting that down-regulation of inflammation by imetit may not be due to direct actions on these cell types. The question remains, how many of the beneficial effects on inflammation and cardiac fibrosis are due to activation of H_3_Rs in the heart versus on presynaptic cardiac sympathetic nerve fibers, especially since H_3_R protein could be detected in some but not all Ang-II-infused hearts. The mechanisms underlying the anti-inflammatory and anti-fibrotic actions of H_3_R activation clearly require further detailed investigation.

Oddly, both H_3_R activation and blockade normalized LV mass and LV/tibia ratio at high doses in mice infused with Ang II. For H_3_R activation with imetit, this appears to be due to reduced LV wall thickness, which although not statistically significant, showed a strong trend toward normalization. This lack of significance could in part be due to there only being a moderate hypertrophic effect in the Ang II model at the time point used in this study (seven days). Conversely, LV wall thickness showed no trend toward being reduced by H_3_R blockade with A331440. Continued wall thickening is somewhat difficult to reconcile with reduced LV mass and LV/tibia ratio, however, there are some possible explanations. One possibility is edema. We reasoned that there may be differences in edema between the imetit and A331440 groups and that less edema may account for reduced LV mass and LV/tibia ratio in the absence of reduced LV wall thickness in the A331440 group. However, we found no difference in LV water content between imetit and A331440 groups (Figure S10). Another alternative is that the heart took on a more spherical shape due to longitudinal shortening in response to A331440, without wall thinning. Unfortunately, we did not perform long-axis imaging of the heart, which could have substantiated this.

In summary, the most significant findings from the present study were (1) the H_3_R is expressed in the mouse heart, more specifically, cardiac fibroblasts, and (2) H_3_R activation was able to reduce cardiac fibrosis and cardiac inflammation. Thus, our findings indicate that the H_3_R has potential as a therapy for cardiac fibrosis, however, further research is required to understand the underlying mechanisms of this anti-fibrotic effect.

## 4. Materials and Methods

### 4.1. Experimental Design

Experiments were performed using 8-week-old male mice (C57BL/6). All mice were purchased through the Kearns Animal Facility at the Kolling Institute of Medical Research and were housed individually under standard environmental conditions and maintained on standard commercial mouse chow and tap water ad libitum. All animal procedures were performed according to the animal ethics protocol: RESP/19/019 approved by the Northern Sydney Local Health District Animal Ethics Committee (AEC) on 21 March 2019. The Ang-II-infusion model was chosen to assess the contribution of the H_3_R to cardiac remodeling. Mice were randomly divided into six groups: (1) saline-infused + saline (*n* = 8); (2) Ang-II-infused (1500 ng/kg/min) + saline (*n* = 9); (3) Ang-II-infused + H_3_R agonist (imetit, 20 mg/kg/day, *n* = 8); (4) Ang-II-infused + H_3_R agonist (imetit, 40 mg/kg/day, *n* = 6); (5) Ang-II-infused + H_3_R antagonist (A331440, 10 mg/kg/day, *n* = 5); and (6) Ang-II-infused + H_3_R antagonist (A-331440, 20 mg/kg/day, *n* = 6). Ang II or saline was delivered continuously via osmotic minipump. For minipump implantation, mice were anesthetized using inhaled isoflurane (2%). Once anesthetized as evaluated by toe pinch reflex, a longitudinal incision was made along the midline of the abdomen. Alzet 7-day minipumps containing either saline or Ang II were peritoneally implanted with minimal disturbance of the bowel. The peritoneum was closed using 5-0 Ethicon chromic gut sutures, whilst the skin wound was closed with surgical staples. Mice received Temgesic (0.1 mg/kg) for post-surgery pain relief. The H_3_R agonist imetit (Tocris, Minneapolis, MN, USA) and the H_3_R antagonist A-331440 (Sigma Aldrich, St Louis, MO, USA) were administered as daily subcutaneous injections. Daily saline injections served as a vehicle control. The experimental period lasted 7 days. At the experimental endpoint, the mice were anesthetized using isoflurane (2%) and euthanized by removal of the heart. The heart was separated into the LV plus septum and the RV. The lungs, LV, and RV were weighed, and tibia length measured. The apex portion of the LV was snap-frozen in liquid nitrogen and stored at −80 °C, whilst the base was fixed in zinc formalin.

### 4.2. Echocardiography

Mice underwent echocardiography (Vevo 3100 Imaging System) at the end of the 7-day experimental period to determine the effects of treatment on cardiac structure and function. Mice were anesthetized with isoflurane (2%) for the procedure. Images were taken at the mid-papillary level with a 30 MHz transducer. Measurements of LVPW and LVID were made using two-dimensional M-Mode in the parasternal short-axis view. LV function was assessed by fractional shortening (FS), stroke volume, and cardiac output calculated as follows:

FS = (LVIDd−LVIDs/LVIDd) × 100,

Stroke volume = LVIDd−LVIDs,

Cardiac Output = stroke volume × heart rate,

where LVIDd and LVIDs represent LV internal diameter in diastole and systole, respectively.

### 4.3. Histology/Immunofluorescence

Collagen volume fraction–picrosirius red staining of fixed LV sections was used to identify fibrillar collagen for assessment of cardiac fibrosis. LV sections were rehydrated in decreasing concentrations of ethanol and then stained with picrosirius red (0.1% Sirius Red F3BA in picric acid). LV sections were then dehydrated in increasing concentrations of ethanol and xylene and mounted with Depex and cover slipped. Stained LV sections were imaged microscopically using a 20x objective. 10 images per LV section were acquired, and each image was analyzed using Image J software. The percentage area of collagen was measured for each image with the average of the 10 images taken to generate the overall collagen volume fraction. Perivascular areas were excluded from collagen analysis.

Mast Cell/Macrophage Labeling–Formalin fixed LV sections of 5 μm thickness underwent rehydration before incubation in boiling citrate buffer for 10 min for antigen retrieval. The sections were labeled with anti-MAC-2 (1:100, Cedarlane, Burlington, VA, USA) to identify macrophages and avidin-(488) (1:100, Life Technologies, Carlsbad, CA, USA) to identify mast cells, following blocking for non-specific binding. MAC-2 labeling was visualized using 568-goat anti-rat secondary antibody (1:100, Life Technologies). All sections were cover slipped with ProLong Diamond containing DAPI (Life Technologies) and visualized under a fluorescence microscope. Macrophage numbers were calculated as the average number of macrophages contained in 5 random fields at 20× magnification. Mast cell numbers were expressed as the total number of avidin positive cells in each LV section (20× magnification).

### 4.4. Isolation and Treatment of Cardiac Fibroblasts

Cardiac fibroblasts were isolated from the LV of male C57BL/6 mice and male Sprague Dawley rats (8 weeks of age) similarly to how we have described previously [1,4,29,30]. Briefly, LV tissue was minced and digested by a series of 5 incubations with 100 ng/µL Liberase TM (Roche, Basel, Switzerland) at 37 °C for 15 min. The cell pellets were resuspended and plated in DMEM-F12 media for mouse fibroblasts and DMEM for rat fibroblasts and supplemented with 10% FBS. Non-adherent cells were removed by washing. Fibroblasts were used after only one passage to minimize changes in phenotype associated with culture. Before treatment, both mouse and rat fibroblasts were serum-starved in DMEM-F12 media for 24 hrs either on gelatin-coated or non-coated 6-well plates and then treated with Ang II (300 nM), TGFβ1 (10 ng/mL), or high glucose (25 mM) for 24 hrs. All treatments were performed in DMEM-F12 (mouse) or DMEM (rat) containing 1.5% FBS except for the high serum positive control (10% serum).

### 4.5. PCR/Sequencing

To initially identify the expression of RNA for the H_3_R in the LV, total RNA from LV tissue (saline and Ang-II-treated) was isolated using the RNeasy Plus Mini Kit (Qiagen, Hilden, Germany) according to the manufacturer’s instructions, quantified using a Nanodrop 1000 UV-Vis spectrophotometer (Thermo Fisher Scientific, Waltham, MA, USA), and then 5 μg of RNA converted to cDNA using Maxima H Minus Reverse Transcriptase (Thermo Scientific). One μL of each cDNA template was amplified with HotstartTaq DNA Polymerase (Qiagen), with the following primers by Morisset et al. [18]: *HRH3* (Forward primer: 5′ TGCTGTATGGGCCTGCCATCCTGAGTTGG 3′, Reverse primer: 5′ CACCATCTTCATGCGCTTCTCCAGGGATGC 3′). The thermal cycling program was performed on a MiniAmp Thermal Cycler (Thermo Fisher Scientific) with an initial denaturation step at 95 °C for 10 min, followed by 35 cycles of 30s denaturation at 94 °C, 30 s annealing at 55 °C, 60s extension at 72 °C, and then a final step holding at 98 °C for 10 min. One μL of each PCR product was then subjected to a second round of PCR using identical conditions. PCR products were resolved on a 2% agarose gel and imaged using a Major Science gel imager. Individual amplicons were isolated by gel extraction using the Wizard® SV Gel and PCR Clean-Up System (Promega, Madison, WI, USA) and identified by Sanger sequencing (Australian Genome Research Facility, Melbourne, Australia). Expression of *Hrh3* was quantified using SensiFAST probe Hi-ROX mastermix (Bioline Australia, Eveleigh, Australia) in combination with mouse *Hrh3* and *Gapdh*-specific Taqman assays (Life Technologies), run on QuantStudio 12K Flex real-time PCR machine (Thermo Fisher Scientific) according to the manufacturer’s instructions. Relative expression of *Hrh3* normalized against *Gapdh* was calculated by the ddCT method using ExpressionSuite Software (Thermo Fisher Scientific).

### 4.6. Protein Analysis

Western blots were used for the identification of H_3_R protein in LV lysates and isolated cardiac fibroblast lysates. Forty micrograms of protein for each sample were mixed in a 1:1 ratio in Lamelli buffer and heated at 90 °C for 15 min. Samples were loaded into precast Mini-Protean TGX gels (BIO-RAD, Hercules, CA, USA) and run at 80 V until separated optimally. Protein was then transferred onto a nitrocellulose membrane. The membrane was checked with Ponceau S solution for successful protein transfer, then blocked with an intercept blocking buffer (TBS) for 1 hr. The membrane was incubated overnight at 4 °C in an H_3_R primary antibody (Thermo Fisher Scientific H_3_R Polyclonal antibody Rabbit IgG, 1:1000). This was followed by incubation with a secondary antibody (Donkey Anti-Rabbit, 1:10,000) for 1 hr at room temperature. The membrane was washed in TBST between each change of antibody and before being imaged on an Odyssey CLx imager. Equal protein loading was determined by GAPDH (Sigma Aldrich, St Louis, MO, USA, 1:1000). Norepinephrine was assessed in LV lysate by ELISA (Aviva Systems Biology, San Diego, CA, USA) as per the manufacturer’s instructions. All samples were run in duplicate.

### 4.7. Hydroxyproline Assay

Collagen production by isolated mouse cardiac fibroblasts was assessed by measuring hydroxyproline levels in the culture media. Briefly, 100 µL of fibroblast cell culture media was incubated with 100 µL of 6N HCl and hydrolyzed at 107 °C for 18 hrs. The samples were dried in a vacuum centrifuge for 3 hrs and reconstituted with 500 μL of dH_2_O. They were then oxidized with 250 μL of chloramine T reagent and developed with 250 μL of Ehrlich’s reagent. Two hundred microliters of each sample, including the standards, were aliquoted into 96 well plates before being read at an absorbance wavelength of 550 nm. All samples were run in duplicate and averaged and hydroxyproline level determined from a hydroxyproline standard curve.

### 4.8. Statistical Analysis

All experimental data were expressed as a mean ± standard deviation (SD) or standard error of the mean (SEM) as appropriate. The statistical significance of the data was determined by *t*-test for comparison of two experimental groups and by one-way analysis of variance (ANOVA) with Tukey post-analysis for comparison of three or more groups. *p* < 0.05 was considered statistically significant.

## Figures and Tables

**Table 1:** Biometrics for A331440 and imetit treatment.

	Saline	Ang II	Ang II + A331440 (LD)	Ang II + A331440 (HD)	Ang II + Imetit (LD)	Ang II + Imetit (HD)
Body Weight (g)	23.5 ± 1.9	25.3 ± 1.7	23.3 ± 1.9	20.3 ± 1.3 **^,††††,‡‡‡^	24.2 ± 1.4	20.9 ± 0.9 *^,††††,‡‡^
LV mass (mg)	77.4 ± 10.0	98.4 ± 8.5 ***	91.2 ± 12.1	76.8 ± 7.3 ^††,‡‡‡^	100.2 ± 12.4 ***	76.8 ± 4.8 ^††,‡‡‡^
LV/Tibia (mg/mm)	4.0 ± 0.6	4.9 ± 0.4 **	4.7 ± 0.7	3.3 ± 0.4 ^††,‡‡‡^	5.1 ± 0.6 ***	3.9 ± 0.2 ^††,‡‡‡^
RV mass (mg)	21.3 ± 1.5	22.2 ± 3.5	26.4 ± 10.0	24.7 ± 3.4	22.8 ± 3.7	23.3 ± 2.5
RV/Tibia (mg/mm)	1.1 ± 0.1	1.1 ± 0.2	1.4 ± 0.5	1.3 ± 0.2	1.2 ± 0.2	1.2 ± 0.1
Lung mass (mg)	115.1 ± 8.5	125.7 ± 10.9	130.2 ± 17.9	130.5 ± 7.2	130.2 ± 10.9	115.8 ± 10.5
Lung/Tibia (mg/mm)	6.5 ± 1.5	6.3 ± 0.6	6.7 ± 0.7	6.6 ± 0.4	6.7 ± 0.5	5.9 ± 0.5

LD = low dose, HD = high dose, BW = body weight, LV = left ventricle, RV = right ventricle. Values are mean ± SD; *n* = 8 for saline, *n* = 9 for Ang II, *n* = 5 for Ang II + A331440 LD, *n* = 6 for Ang II + A331440 HD, *n* = 10 for Ang II + imetit LD, *n* = 6 for Ang II + imetit HD; * *p* < 0.05 vs. saline; ** *p* < 0.01 vs. saline; *** *p* < 0.001 vs. saline; ^††^ *p* < 0.01 vs. Ang II; ^††††^ *p* < 0.0001 vs. Ang II, ^‡‡^ *p* < 0.01 vs. corresponding LD group; ^‡‡‡^ *p* < 0.001 vs. corresponding LD group.

## Supplementary Materials

Supplementary Materials can be found at https://www.mdpi.com/1422-0067/21/24/9757/s1.

## Abbreviations

H_1_R	Histamine 1 receptor
H_2_R	Histamine 2 receptor
H_3_R	Histamine 3 receptor
H_4_R	Histamine 4 receptor
MESA	Multi-ethnic Study of Atherosclerosis
Ang II	Angiotensin II
LV	Left ventricle
RV	Right ventricle
LVIDd	Left ventricular internal diameter in diastole
LVPWd	Left ventricular posterior wall thickness in diastole
