# Supplementary material for: The Histamine 3 Receptor Is Expressed in the Heart and Its Activation Opposes Adverse Cardiac Remodeling in the Angiotensin II Mouse Model
Source: Int J Mol Sci. 2020 Dec 21;21(24):9757. doi: 10.3390/ijms21249757 (PMC7767352; doi:10.3390/ijms21249757)
Supplement: Supplementary file 1 [file ijms-21-09757-s001.pdf]

Supplementary Materials

The Histamine 3 Receptor is Expressed in the Heart and its Activation Opposes Adverse Cardiac Remodeling in the Angiotensin II Mouse Model

Samuel L. McCaffrey[1,2], Grace Lim[1], Martyn Bullock[1], Ainsley O. Kasparian[1,3], Roderick Clifton-Bligh[1], William B. Campbell[4], Alexander Widiapradja[1,2], Scott P. Levick*[1,2]

[1]Kolling Institute of Medical Research, Royal North Shore Hospital, St Leonards, Australia

[2]Faculty of Medicine and Health, The University of Sydney, Sydney, Australia

[3]School of Life Sciences, University of Technology Sydney, Sydney, Australia

[4]Pharmacology and Toxicology, Medical College of Wisconsin, Milwaukee, USA

Short Title: Histamine 3 Receptor and Cardiac Remodeling

Corresponding Author:

Scott P. Levick, Ph.D., FAHA

Faculty of Medicine and Health

The University of Sydney

Kolling Institute of Medical Research

St Leonards, NSW 2006

Telephone: +61 2 99264911

Email: scott.levick@sydney.edu.au

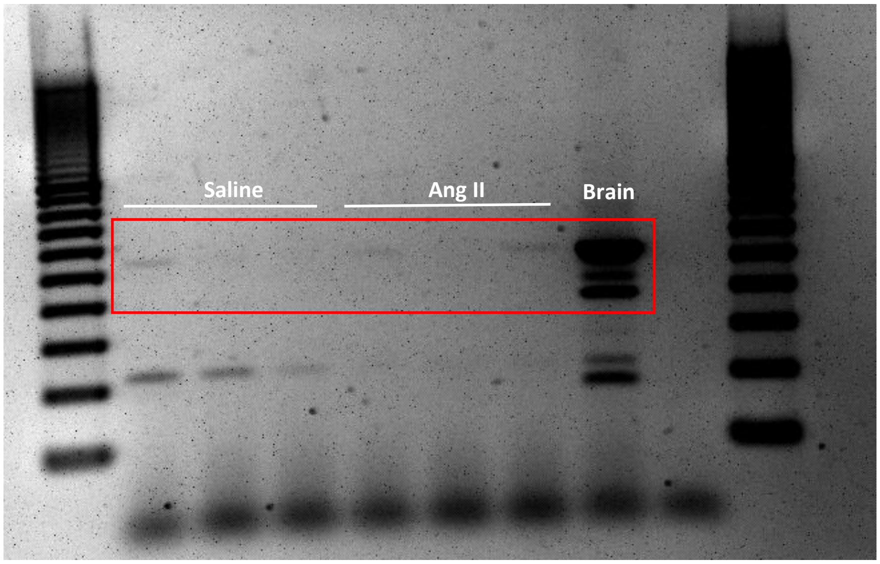

Figure S1. Full PCR gel that appears in cropped form in Figure 1A. Shows the ~530 bp product for the left ventricle from Saline and Ang II-infused mice, as well as the ~530, 430 and 400 bp products for the mouse brain (red box).

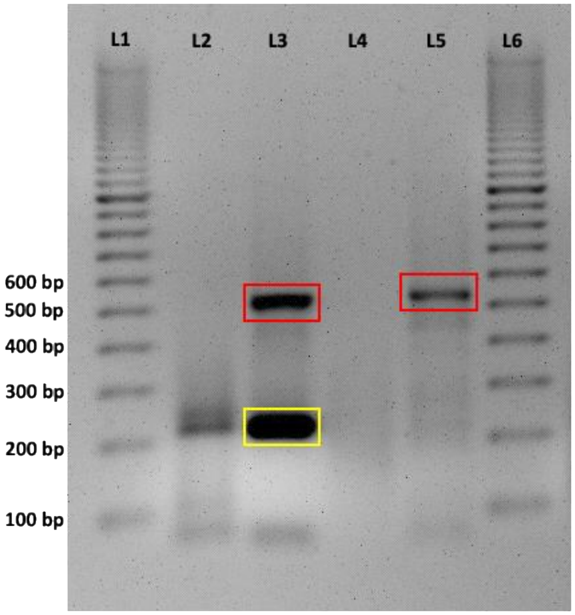

Figure S2. Full PCR gel showing the ~530 bp product (red box) from the Ang II LV (L3) and brain (L5) that were subsequently purified and identified by Sanger sequencing as a 99% match for the mouse H_3R. The ~200 bp product (yellow box) from the LV was also purified, sequenced and identified as a 98% match for mouse endoplasmic reticulum chaperone SIL1 homolog. L1 = ladder, L2 = saline LV, L4 = empty, L6 = ladder.

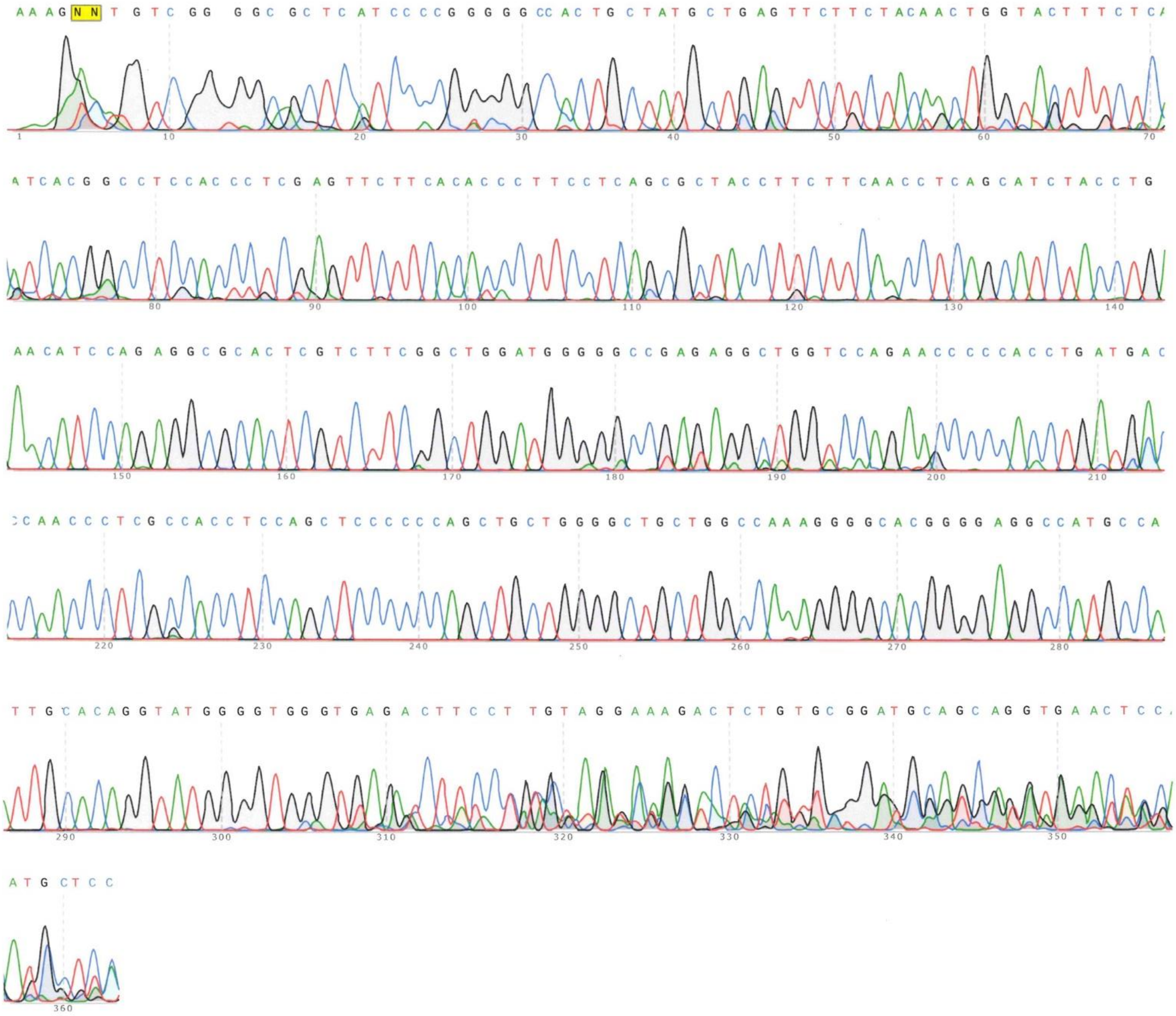

Figure S3. (A) Electrophoretogram identifying the amplicon of the 530 bp PCR product identified as a 99% match for *mus musculus histamine 3 receptor* – forward primer.

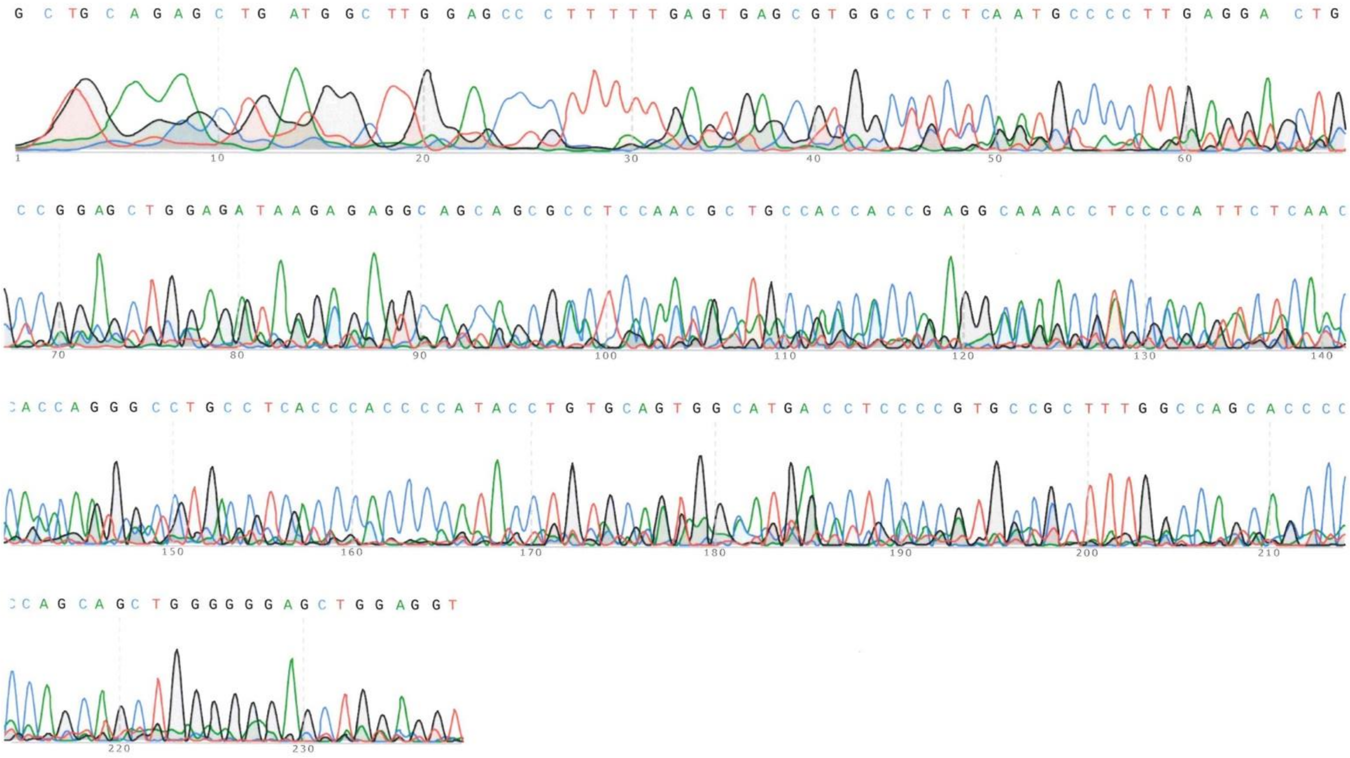

Figure S3. (B) Electrophoretogram identifying the amplicon of the 530 bp PCR product identified as a 99% match for *mus musculus histamine 3 receptor* – reverse primer.

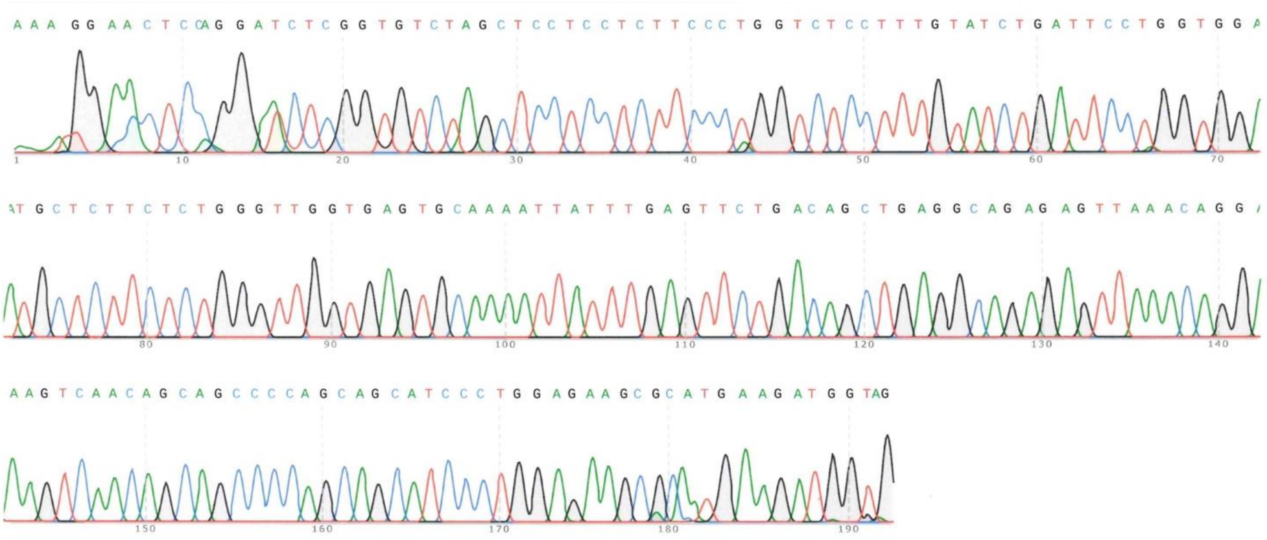

Figure S3. (C) Electrophoretogram identifying the amplicon of the 200 bp PCR product identified as a 98% match for *mus musculus endoplasmic reticulum chaperone SIL1 homolog* – forward primer.

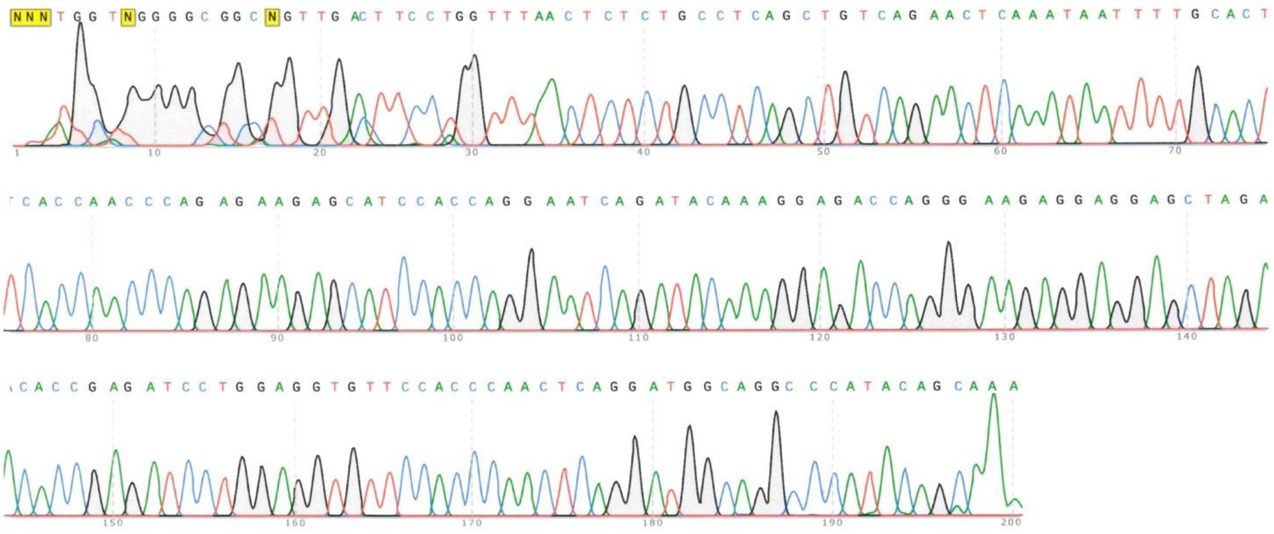

Figure S3. (D) Electrophoretogram identifying the amplicon of the 200 bp PCR product identified as a 98% match for *mus musculus endoplasmic reticulum chaperone SIL1 homolog* – reverse primer.

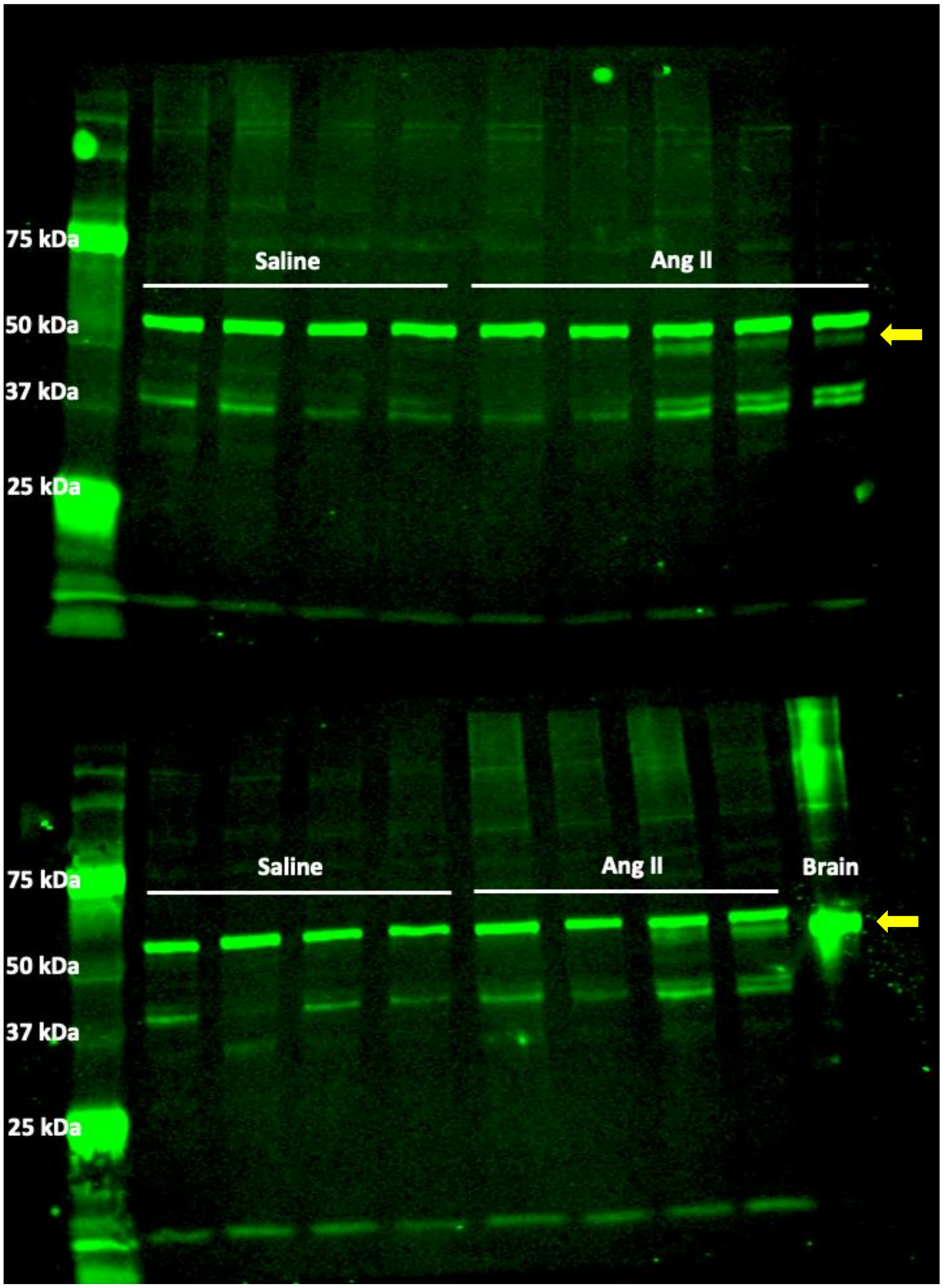

Figure S4. Image of full-length western blots for the H_3R (yellow arrow) in LV lysate from saline and Ang II-infused mice that appears in cropped form in Figure 1C.

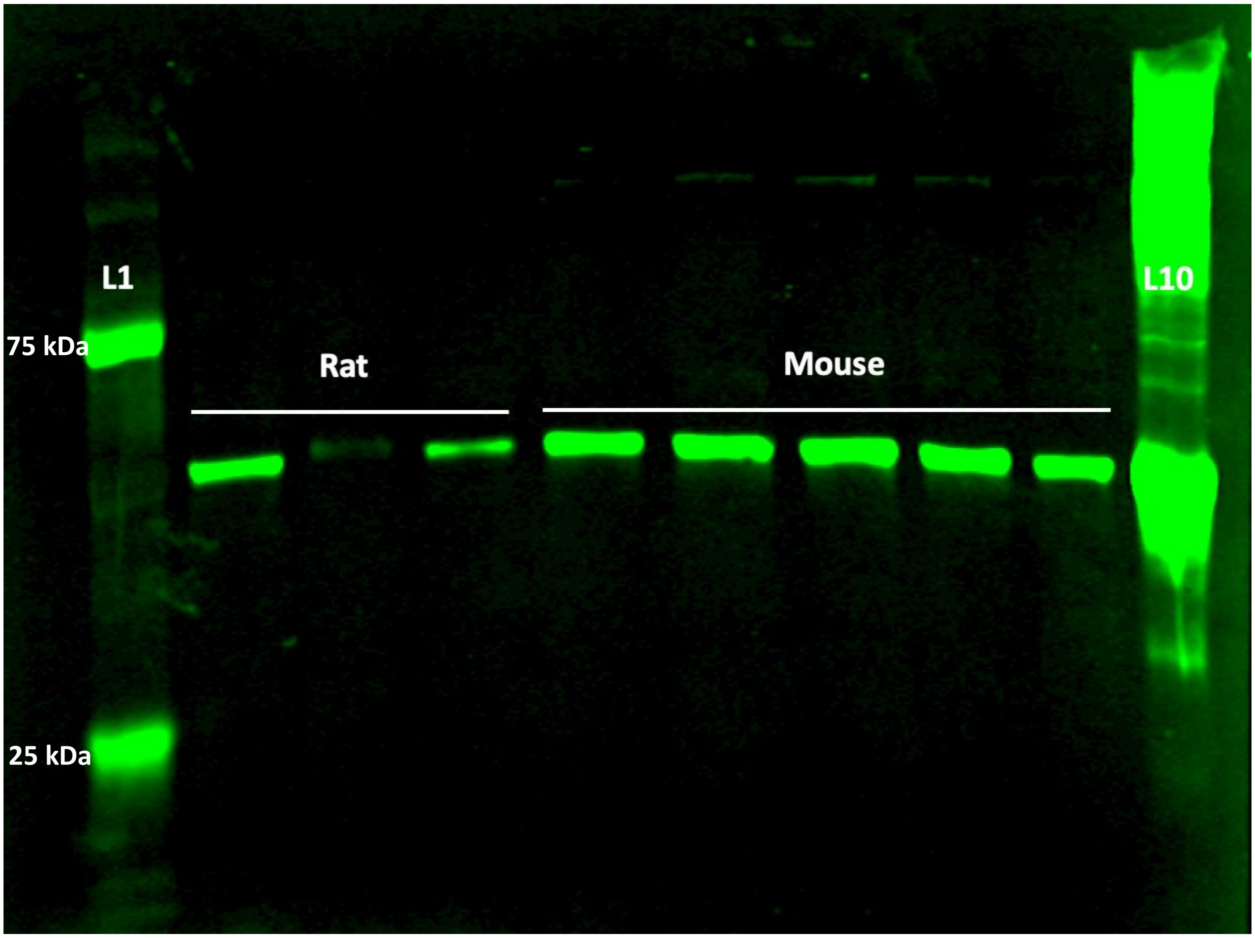

Figure S5. Image of full-length western blot for the H_3R in protein lysate from isolated rat and mouse cardiac (myo)fibroblasts that appears in cropped form in Figure 2A. Lane 1 (L1) is the molecular weight ladder. Lane 10 (L10) is brain extract as a positive control for the H_3R. Lane specifics are contained in the figure legend for Figure 2.

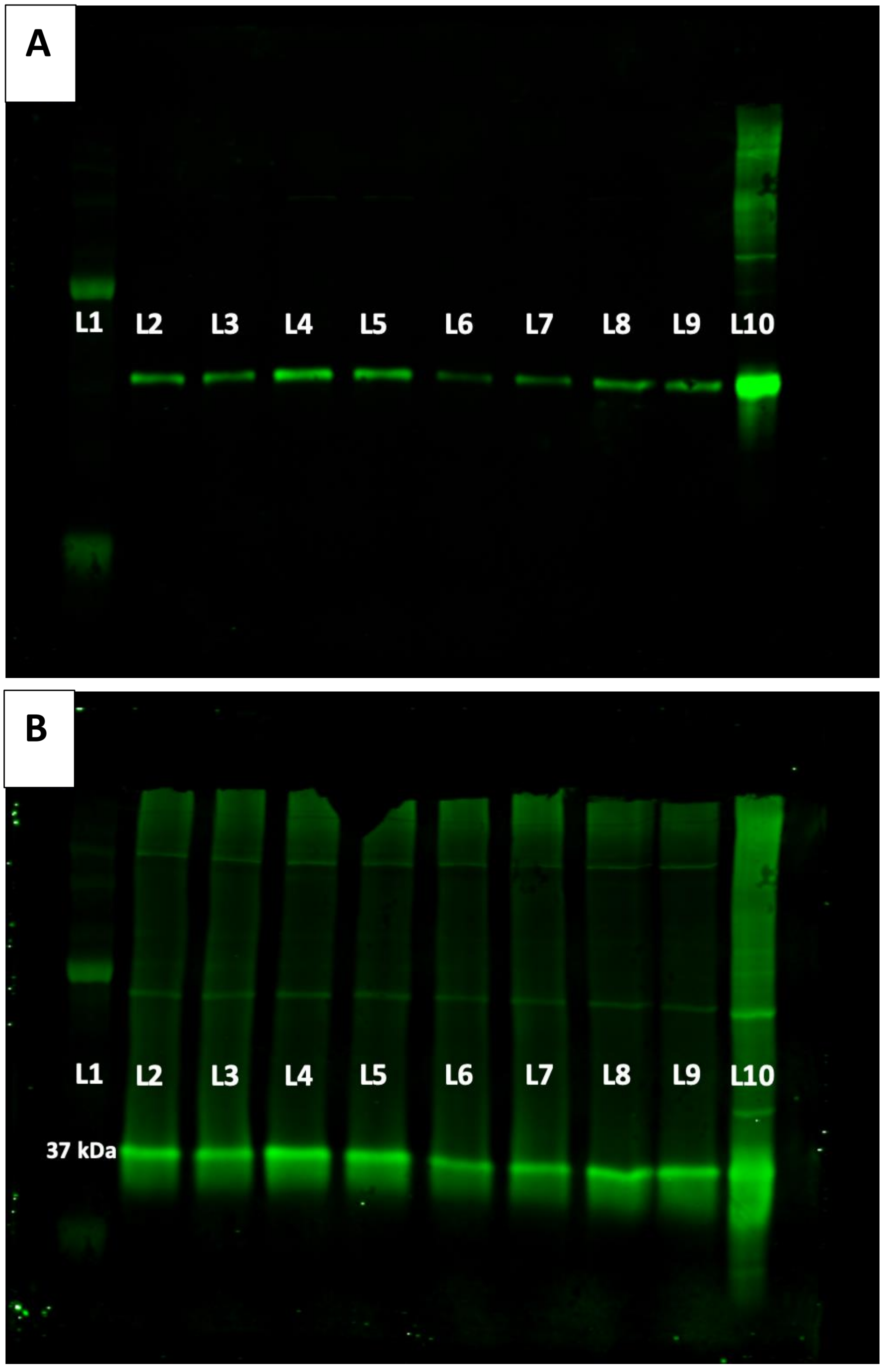

Figure S6. Images of full-length western blots for **(A)** the H_3R and **(B)** GAPDH in control cardiac fibroblast and cardiac fibroblasts treated with Ang II (300 nM) that appears in cropped form in Figure 2B. Lane 1 (L1) = molecular weight ladder, L2/3 = control, L4/5 = Ang II treated, L6/7 = control, L8/9 = Ang II treated, L10 = brain extract as a positive control for H_3R.

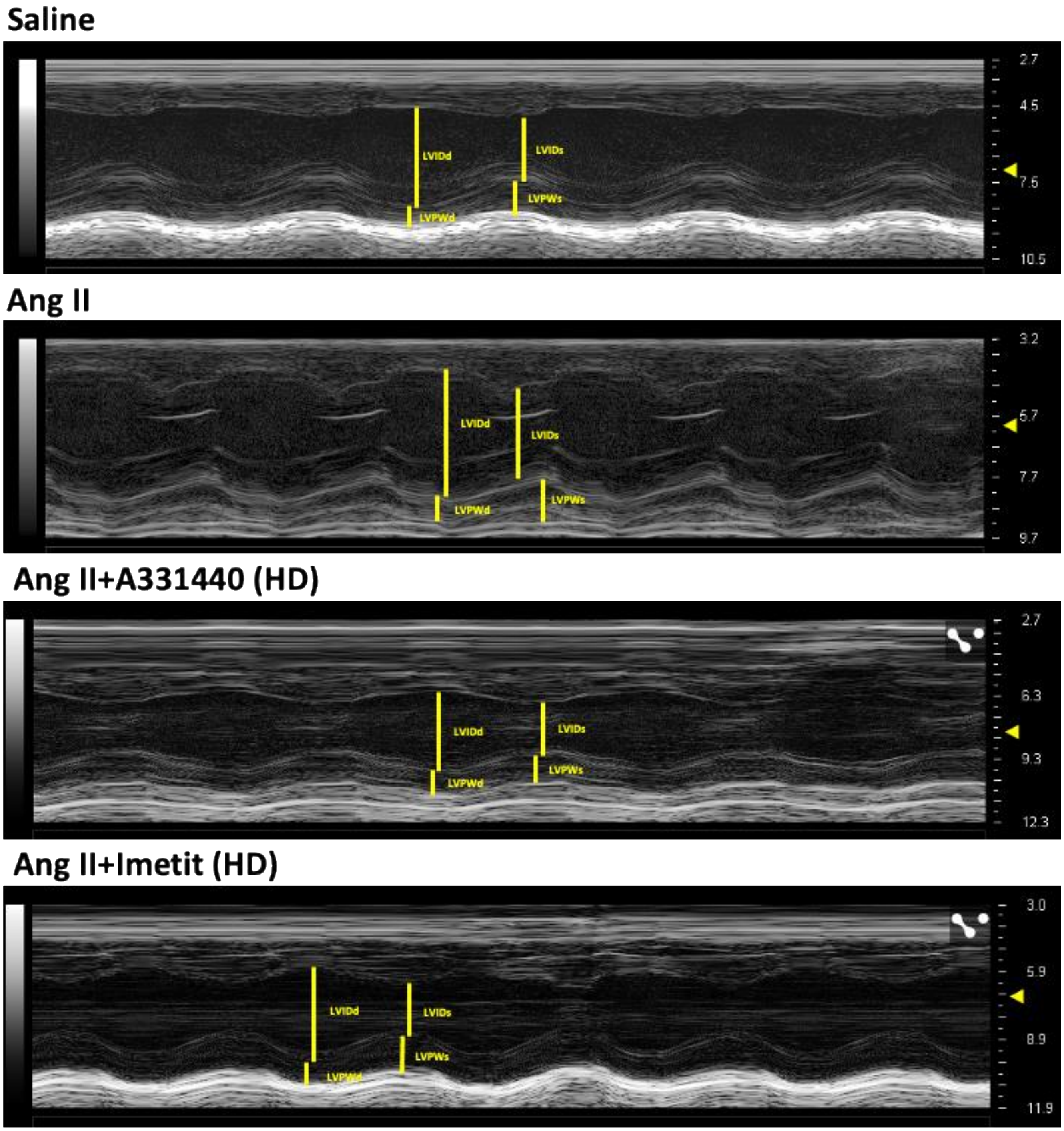

Figure S7. Example echocardiographic images for Saline, Ang II, Ang II+A331440 (HD) and Ang II+Imetit (HD) groups.

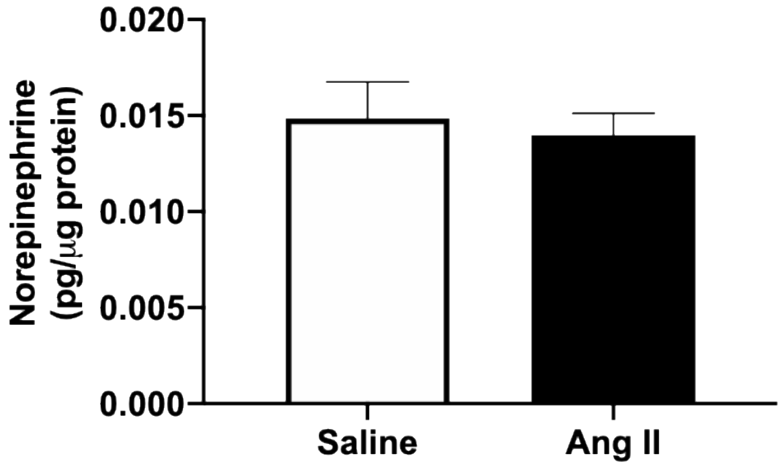

Figure S8. Norepinephrine levels from Saline and Ang II-infused mouse LV lysates. Data are mean ±SEM.

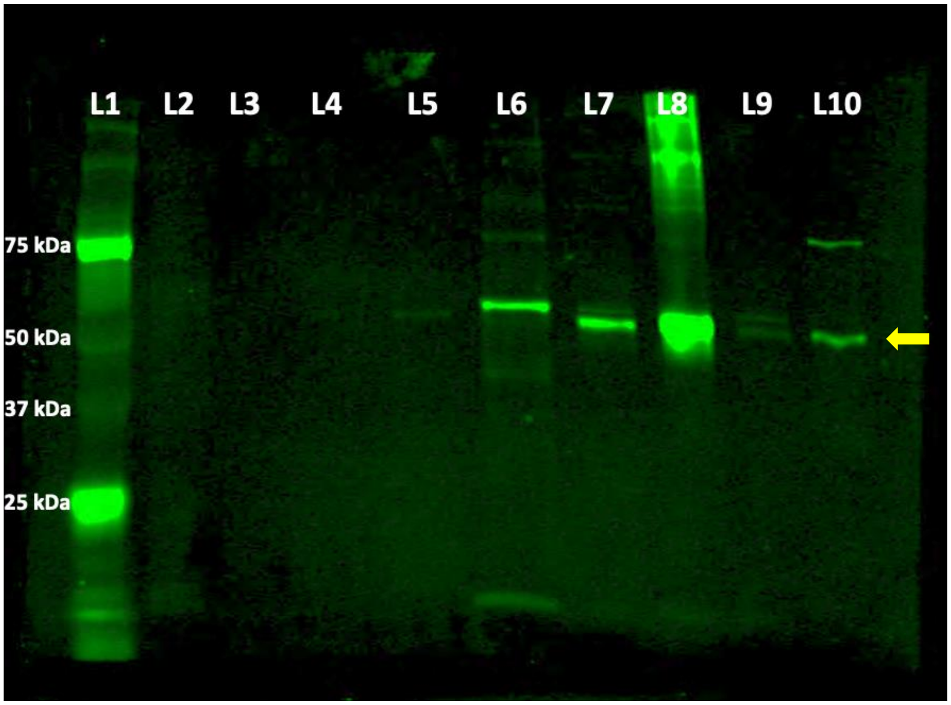

Figure S9. Western blot for the H_3R (50 kDa, yellow arrow). L1 - Ladder, L2 - Bone marrow-derived mast cells, L3 - Bone marrow-derived macrophages, L4 - H9c2 cells (immature cardiomyocytes), L5 - Cardiac fibroblasts, L6 - LV, L7 - Lung, L8 - Brain, L9 - Thyroid, L10 - Thyroid cell line (NThy)

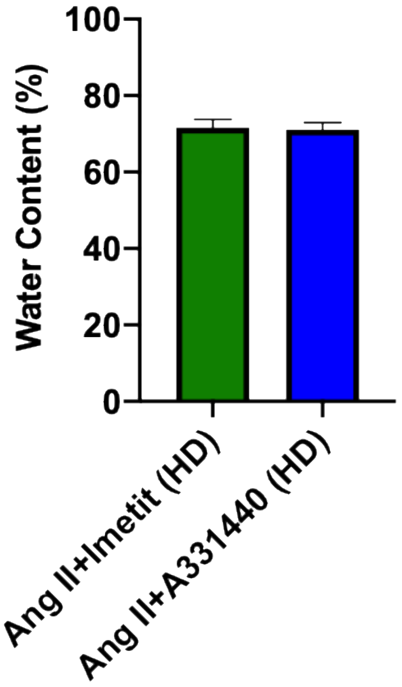

Figure S10. LV water content following Ang II-infusion for high dose (HD) Imetit and A331440 groups. Data are mean ±SD.
